# 3D Printing Resins Involving Exclusively Bio-Based Compounds—Can Precursors for Commercial Vat Photopolymerization Comprise Only Biobased Molecules?

**DOI:** 10.1021/prechem.6c00065

**Published:** 2026-07-30

**Authors:** Vojtěch Jašek, Radek Přikryl

**Affiliations:** Institute of Materials Chemistry, Faculty of Chemistry, 48274 Brno University of Technology, 61200 Brno, Czech Republic

**Keywords:** vat photopolymerization, biobased resins, reactive diluent, photoinitiation, 3D printing

## Abstract

Vat photopolymerization exemplifies one of the most attractive and prospective processing approaches, producing unique prototypes, specifically functional systems, or biocompatible products in the next generation of biobased materials. This work summarizes and presents specific strategies for developing chemical solutions for three-dimensional (3D)-printable resins derived exclusively from biobased compounds. The photocurable systems used in additive manufacturing require a primary reactive resin precursor. Usually, rheological modifications that regulate the flow characteristics of the polymerizable main system require the use of reactive diluents, which are low-molecular-weight, low-viscosity, and polymerizable. Such systems often exhibit excessive volatility, resulting in environmental issues such as increased VOCs or hazardous chemical character. This overview presents and describes the main reactive resin precursors, based on bioderived polyesters and terpene-based systems, along with fully biobased reactive diluents synthesized from biotechnological products (itaconic acid) and other sources (biophenols). Lastly, the partly and fully biobased photoinitiators, produced from sources, such as riboflavin, coumarin, or curcumin, are introduced as an alternative to the highly hazardous and toxic commercial photoinitiators, such as BAPO and TPO, which pose serious health hazards. Fully biobased precursors stand in this perspective as substances obtainable from alternative feedstocks to petroleum-based ones (e.g., biotechnological production, extraction from biomass, and reformulation of complex substances). This work reviews the reported options and experimental findings and suggests the most promising pathways to environmentally friendly vat photopolymerization resins.

## Graphical abstract

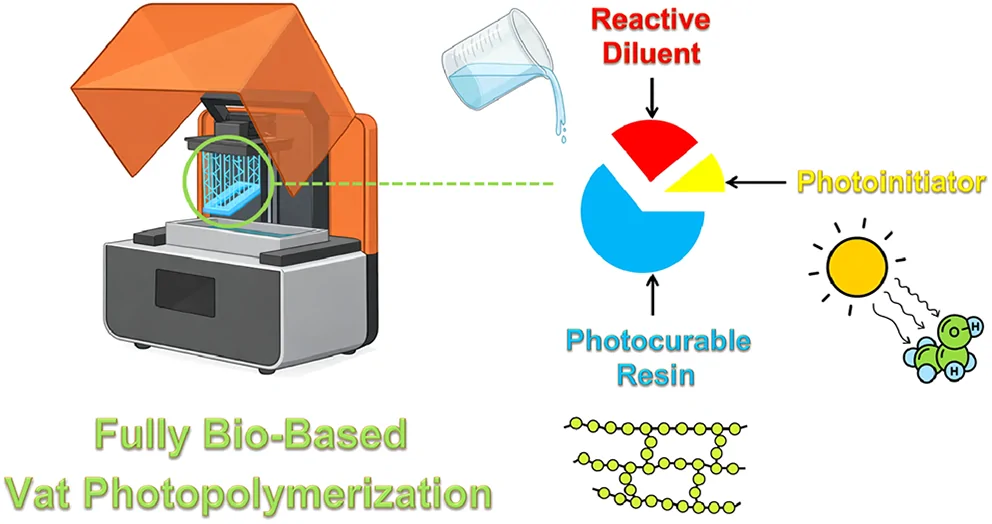

## 1 Introduction

Additive manufacturing is widely spreading technology in various application fields and also in the commercial sphere due to a high level of a precise and detailed prototypes’ production,^1^ unlimited opportunities for art sector,^2^ a higher sustainability factor compared to the opposite subtractive manufacturing (involving material wasting procedures, such as cutting, drilling, or grinding),^3^ and numerous different approaches toward mono- and multicomponent materials.^4^ Three-dimensional (3D) printing is an alternatively used term for additive manufacturing, describing a process creating three-dimensional products (prints) layer-by-layer, which minimizes the material waste and maximizes the efficiency of the creating process.^5^ Since the polymer-forming sector involves numerous different strategies toward the final materials, 3D printing includes various strategies comprising various materials and precursors.^6^ The processing of linear and fusible systems, such as polylactic acid (also known as polylactide) (PLA),^7^ acrylonitrile butadiene styrene (ABS),^8^ or poly­(ethylene terephthalate glycol)-modified (PETG),^9^ typically involves material extrusion as the primary processing approach.^10^ Fused deposition modeling (FDM) and fused filament fabrication (FFF) are particular 3D printing techniques that use linear polymers.^11,12^ Similarly to the previous route processing solid-phase linear polymers, 3D printing approaches, such as selective laser sintering (SLS), selective laser melting (SLM), or electron beam melting (EBM), use fusible macromolecular systems or metal starting systems.^13–15^ However, these strategies work with precursors in powder form rather than extruded filaments. This additive manufacturing process, called powder bed fusion, uses a thermal energy source to selectively transform powder precursors into the final prints.^16,17^ Apart from the polymer materials (mostly polyamides (nylon-11 (PA 11), nylon-12 (PA 12))^18,19^ and thermoplastic polyurethane (TPU)),^20^ primarily processed via SLS, powder material 3D printing also uses metal systems (stainless steel, titanium, or aluminum), which are processed by SLM and EBM.^21,22^ The aforementioned polymer and metal processing approaches can arguably transform fully biobased materials (such as polylactic acid) or commonly mined metals into printed prototypes.^23,24^ Therefore, 3D printing strategies using solid-phase starting materials can produce exclusively biobased prints, and the final applications and properties must be engineered and specified according to the customers’ requirements.^25^

Vat Photopolymerization exemplifies an entirely different manufacturing approach compared to the previously introduced and described techniques. This manufacturing strategy involves two terms. “Vat” stands for a tank or tub filled with a liquid-form resin (unlike the previously listed linear polymers/metals 3D printing approaches). “Photopolymerization” refers to the approach toward obtaining a solid-phase printed prototype from a liquid precursor.^26,27^ This method uses an irradiation source (usually blue light and UV irradiation) to selectively cure the polymerizable liquid precursor in a vat, forming thermoset polymer prints.^26,27^ Several types of vat photopolymerization are distinguished by the irradiation source used. Stereolithography (SLA) is the originally proposed method, using laser irradiation to cure the precursor point by point with a monochromatic, selective source.^28^ This technique is exceptionally precise but slow at the same time.^29^ Digital light processing (DLP) is a novel, enhanced vat photopolymerization approach that uses a digital projector to expose an entire layer or printed area at once.^30^ This strategy increases the printing speed compared to SLA tremendously.^31^ Masked stereolithography (MSLA) also uses a screen, particularly a liquid-crystal display (LCD), to mask the exposure irradiation source, forming an entire printed layer similar to DLP.^32^ MSLA instrumentation often results in lower acquisition costs; however, DLP provides higher accuracy than MSLA (according to experimental investigations).^33^ Two-photon polymerization approach (2PP) exemplifies an advanced and highly studied additive manufacturing strategy, involving an enhanced irradiation source and opportunities for printing in micro- and nanoscale.^34^ The two-photon absorption refers to the process of simultaneous interaction between the photoinitiator molecule and two low-energy photons (possessing a wavelength of 600–800 nm).^35^ Since the photoinitiator absorbs two photons, the eventual energetic exposure multiplies, and the polymerization reaction initiates directly at the focal point. This approach requires extremely precise optical hardware to focus two irradiation sources into a single focal point, and a highly reactive, polymerizable liquid precursor that can quickly initiate curing (typically using acrylates rather than methacrylates).^36^ The long processing times, complicated and expensive functional hardware, and microscope-requiring observation highlight the limitations of this method, seeking its applicability in the biomedicinal sector^37^ or micro optics/devices.^38^

The initially introduced linear-polymer-based 3D printing strategies have been extensively investigated in the published literature from a sustainable perspective.^39–41^ Pure PLA 3D printing filaments are commercially available, offering an opportunity for sustainable additive manufacturing using an entirely biobased system.^42^ Additionally, several experimental studies incorporated several polyhydroxyalkanoates (PHAs) into additive manufacturing, mostly in suitable blends with PLA systems due to the complicated solo processability of most PHAs.^43,44^ Commonly, fully biobased polyesters, such as PLA or poly­(3-hydroxybutyrate) (P3HB), exhibit lower thermal stability than fossil-based systems, such as ABS or PETG.^45^ Moreover, the 3D printing systems from biobased polyesters tend to be hydrolyzable and unstable.^46^ Nevertheless, these properties are advantageous for a potential application in the medicinal sector or drug delivery, where biocompatibility and biodegradability play a key role.^47,48^

The vat photopolymerization approach in additive manufacturing faces a much more complex context for the production of fully biobased prototypes and prints (see Figure 1). As described, this strategy requires a reactive, liquid-form, low-viscosity polymerizable system capable of efficiently and selectively forming a cured center or layer. Such systems are commonly based on either radically polymerizable acrylates and methacrylates or cationically polymerized epoxides.^49,50^ Methacrylates are commercially used due to their relatively low manipulation hazards and customer-friendly processing, compared to acrylate systems, which typically exhibit higher health hazards and generally higher volatility (especially in low-molecular forms).^51^ However, both acrylates and methacrylates should be handled with extreme care due to the several irritation hazards and acute toxicity after intake. Such systems react extremely fast (usually layers of 50 μm thickness printed by the DLP can be cured in 3–5 s),^52^ and the spontaneous polymerization (triggered by the sunlight) is minimized due to the oxygen inhibition (the molecular oxygen in the atmosphere (triplet biradical) reacts with the spontaneously formed radical centers and inactivates (terminates) them).^53,54^ The common epoxide-reactive precursors for vat photopolymerization exhibit multiple disadvantages compared to acrylates/methacrylates. Their polymerization process is significantly slower than that of the radically polymerizable system (due to cationic polymerization).^55^ Systems containing the reactive epoxy functional groups are sensitive to moisture (water molecules react with the epoxy group via “ring opening” nucleophilic substitution, decreasing the reactivity of the epoxy precursor and degrading its quality).^56^ Additionally, since epoxy resin for 3D printing is commonly derived from bisphenol A, particularly bisphenol A diglycidyl ether (DGEBA) or hydrogenated bisphenol A (HBPA), the long-term health risks may include potential carcinogenicity or endocrine disruption.^57^

**Figure 1:**
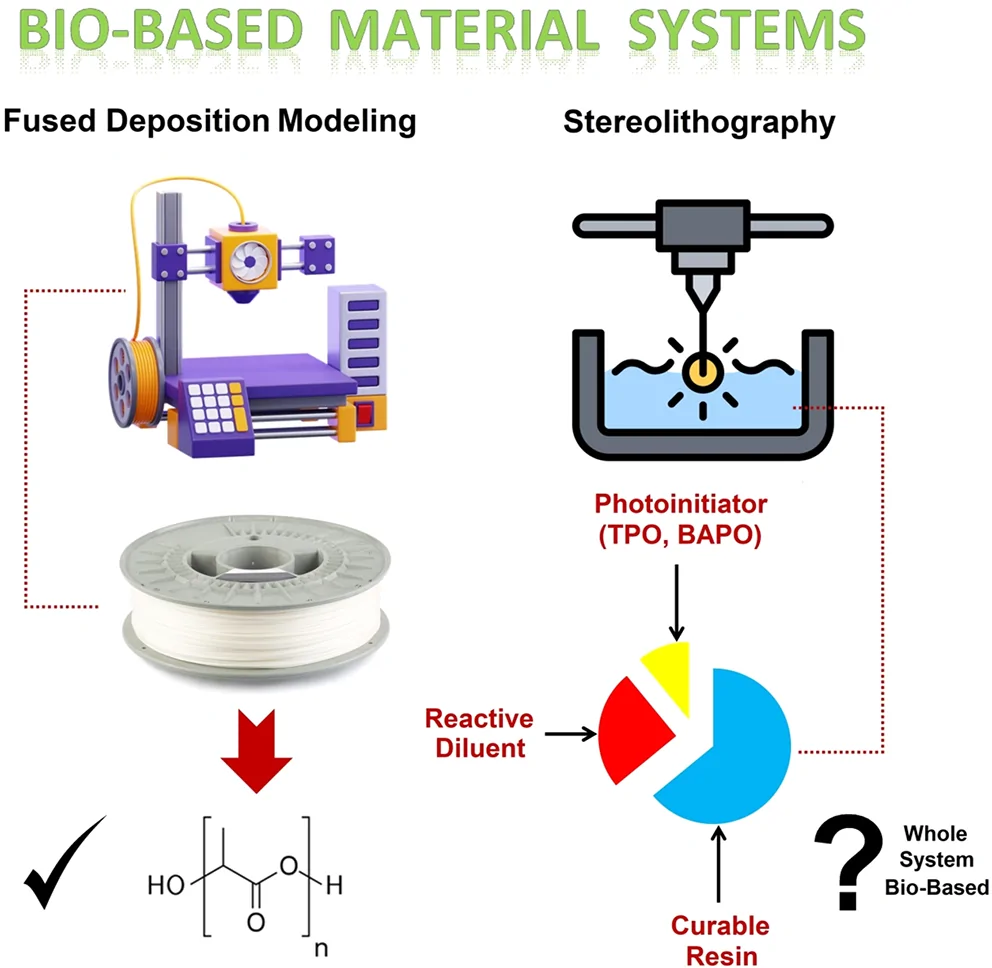
Fully biobased systems used in additive manufacturing were successfully investigated in linear polymer-involving processes (such as fused deposition modeling, FDM). Entirely biobased systems are currently being studied for application in reactive liquid systems for stereolithography (SLA).

Next to the fossil-based resins regularly used in additive manufacturing, many experimental investigations focused on reactive liquid systems and partly biobased polymerizable systems derived from various compounds. Many biobased sources containing modifiable functional groups, such as unsaturated triacylglycerides,^58^ terpenes,^59^ lignin derivatives,^60^ and the naturally occurring polyols,^61^ exemplify starting biobased molecules and systems for 3D-printed materials. The final reactive precursors derived from the mentioned compounds are only partially biobased, due to the commonly used acrylic/methacrylic acids or their derivatives, which are used to functionalize the starting systems to ensure reactivity and suitability for additive manufacturing.^62^ Several strategies were investigated for the synthesis of the curable vegetable oils,^58^ modified monoesters of polymerizable fatty acids,^63^ functionalized polyols, such as glycerol or isosorbide,^64,65^ various lignin derivatives, such as vanillin, vanillin alcohol, and cinnamyl alcohol.^66,67^ Besides the main curable precursors synthesized from biobased sources and functionalized by suitable acrylic/methacrylic derivatives, such as anhydrides, acyl halides, or the pure acid,^68–70^ several reactive diluents, serving as the rheology modifiers, were experimentally investigated in the available literature sources, namely, tetrahydrofurfuryl acrylate (THFA)^71^ or isobornyl acrylate/methacrylate (IBOA/IBOMA).^72,73^ Naturally, practically all of the commercially used liquid precursors for additive manufacturing contain artificially synthesized photoinitiators, such as diphenyl­(2,4,6-trimethylbenzoyl)­phosphine oxide (TPO) or phenylbis­(2,4,6-trimethylbenzoyl)­phosphine oxide (BAPO, Irgacure 819).^74^ Such multicomponent reactive liquid systems were previously repeatedly produced from biobased sources, but the petroleum-based compounds were irreplaceable for several functional modifications and the photoinitiator fabrication.

This work provides a comprehensive overview of the mandatory components for polymerizable liquid 3D printing precursors synthesized entirely from biobased sources. The main material-forming systems, the essential additives and reactive precursors, and even the performing biobased photoinitiators are summarized, described, and evaluated in this article. Is there a possibility of developing a fully biobased, polymerizable, sufficiently reactive, appropriately viscous, low- or nontoxic, nonvolatile, and affordable system as an alternative to the currently used petroleum-based curable systems?

## 2 Photocurable Resins

The specific systems developed for 3D printing have extremely limited opportunities to comprise exclusively biobased sources while remaining suitable for additive manufacturing. Vat photopolymerization, applied to the processing of thermoset-cured materials, involves a specific polymerization that yields a solid-phase final structure. Two main strategies are mostly applied—radical polymerization and cationic polymerization.^75,76^ Radical polymerization involves three stages: initiation, propagation, and termination.^77^ Alkene functional groups typically undergo this type of reaction after a particular initiation involving thermal (peroxides), photo (BAPO, TPO), or UV initiator (benzophenone).^78,79^ The most common substances and functional groups participating in this thermoset-forming strategy include acrylates, methacrylates, styrene, or vinyl esters.^80,81^ Cationic polymerization also involves the three stages previously mentioned during polymer formation; however, different types of initiators are used, such as Lewis acids (AlCl_3_, TiCl_4_, SnCl_4_) or photoinitiators, iodonium, and sulfonium salts.^82–84^ The difference between radical and cationic polymerization lies in the intermediates formed. During radical polymerization, free radicals, which contain an unpaired valence electron, are extremely reactive. Such intermediates react vigorously with the alkene functional group due to its thermodynamic stability—the formed radical reacts with the alkene through an addition mechanism (the double bond converts to a single bond), forming a much more stable radical from a thermodynamic standpoint. During propagation, the radical incorporated into the former alkene group tends to react with another monomer double-bond-containing structure to form an even more stable radical system. Finally, the termination step inactivates the highly reactive free radical by pairing it with another radical, resulting in a stable final compound.^85^ Cationic polymerization does not involve free radicals in the reaction; instead, the reactive ionic structures are generated and attract opposite species. During cationic polymerization, a highly reactive cation must be generated (via protonation or suitable photoinitiation) and then seek a suitable nucleophile to propagate the polymerization. Atoms and structures with extra unbounded valence electron pairs are ideal candidates for such propagation. Epoxy functional groups are commonly used for cationic polymerization due to their exceptional reactivity and the two valence electron pairs on the oxygen atom. First, the epoxy group is protonated, leading to epoxy “ring opening” and the formation of a carbocation on the carbon backbone. This unstable species seeks a suitable nucleophile, another epoxy-bonded oxygen atom, and forms an aliphatic ether linkage. The termination of cationic polymerization involves reaction with a counteranion, a suitable terminal nucleophile, or an impurity.^86,87^

Regarding the opportunities to ensure entirely biobased compounds and systems for vat photopolymerization 3D printing, several published works report precursors declared as “fully biobased,” but they contain fossil-based functional derivatives to ensure their utility for additive manufacturing. Rosa et al.^88^ synthesized and presented 3D printing of acrylate epoxidized soybean oil (AESO) modified by a biobased reactive diluent, isobornyl methacrylate (IBOMA). The authors presented the experimental results incorporating different ratios of the mentioned systems (10–50 wt % of IBOMA content in AESO as a reactive diluent and properties enhancer). Although the system with 50 wt % IBOMA achieved mechanical properties similar to those of the commercial, entirely fossil-based resin (Anycubic standard). The acrylate functional groups in AESO and the methacrylate content in IBOMA exclude this system from consideration as a fully biobased resin system. A similar approach was demonstrated by Colucci et al.,^89^ who synthesized, investigated, and evaluated systems based on AESO, IBOMA, and tetrahydrofurfuryl acrylate (THFA). They also involved natural fillers in their resins—industrial waste corn (GTF) and wine (WPL-CF). Although many interesting and highly performing 3D-printed prototypes were illustrated and produced, the presented system cannot be considered fully biobased due to the incorporation of methacrylate and acrylate derivatives.

Besides the partial fossil-based content in the modified triacylglycerides, which excludes these systems from the group of fully biobased compounds, the modified vegetable oils and their analogues pose several challenges for specific applications. Earlier, high viscosity levels were mentioned, requiring reactive diluents for an optimal applicability in additive manufacturing. Apart from the flow characteristics, the suboptimal photocuring activity of large acrylated/methacrylated triacylglycerides represents a significant disadvantage for vat photopolymerization, increasing total working times (especially for methacrylated oils).^58^ Finally, the mechanical properties of photocured oils typically do not reach excellent levels.^90^ The applicable advantage lies in their worldwide availability, straightforward chemical synthesis, and nonvolatility, which is also significant for health and environmental safety.^91,92^

Next to modified vegetable oils used in vat photopolymerization, several other biobased compounds and systems have been reported as alternatives to entirely petroleum-based resins but cannot be considered “fully biobased.” Chin et al.^93^ demonstrated two lignin-derived liquid precursors suitable for additive manufacturing, namely, 4-propylguaiacyl acrylate (4-pGA) and syringyl methacrylate (SMA) (see Figure 2). Such structures exemplify novel and interesting systems from a waste-volatilization perspective. However, the authors demonstrated that their products are synthesized using acryloyl chloride to modify the free hydroxyl groups of 4-propylguaiacol and syringol, with triethylamine (TEA) and dichloromethane as the catalyst and supporting solvents, respectively. Maturi et al.^94^ demonstrated the most promising fully biobased polyester oligomer system derived from itaconic acid (a fully biobased radical polymerizable compound), glycerol, vanillic acid (derived from lignin), and 1,3-propanediol (see Figure 3). Such a polyester structure may retain an entirely biobased system, but polyester systems typically exhibit excessive viscosity, which complicates their utility for vat photopolymerization. Excessive rheological profiles pose an obstacle to the efficient operation of common vat 3D printers because of suboptimal flow within the supporting printing platform. The printing hardware must be meticulously set to operate at extremely low working rates to ensure a successful processing. Therefore, several reactive diluents are usually used. In the aforementioned investigation, the authors synthesized highly biobased, innovative itaconic acid and citric acid derivatives incorporating 2-hydroxyethyl methacrylate (HEMA), which is not fully biobased. In the described investigation, HEMA was linked to itaconic and citric acid via Fischer esterification, forming low-viscosity diluents that accounted for nearly 50 wt % of the proposed 3D-printed precursor. Although the published study reports a fully biobased polyester substance, the assisting partly fossil-based compounds must have been incorporated to achieve successful vat photopolymerization.^95^

**Figure 2:**
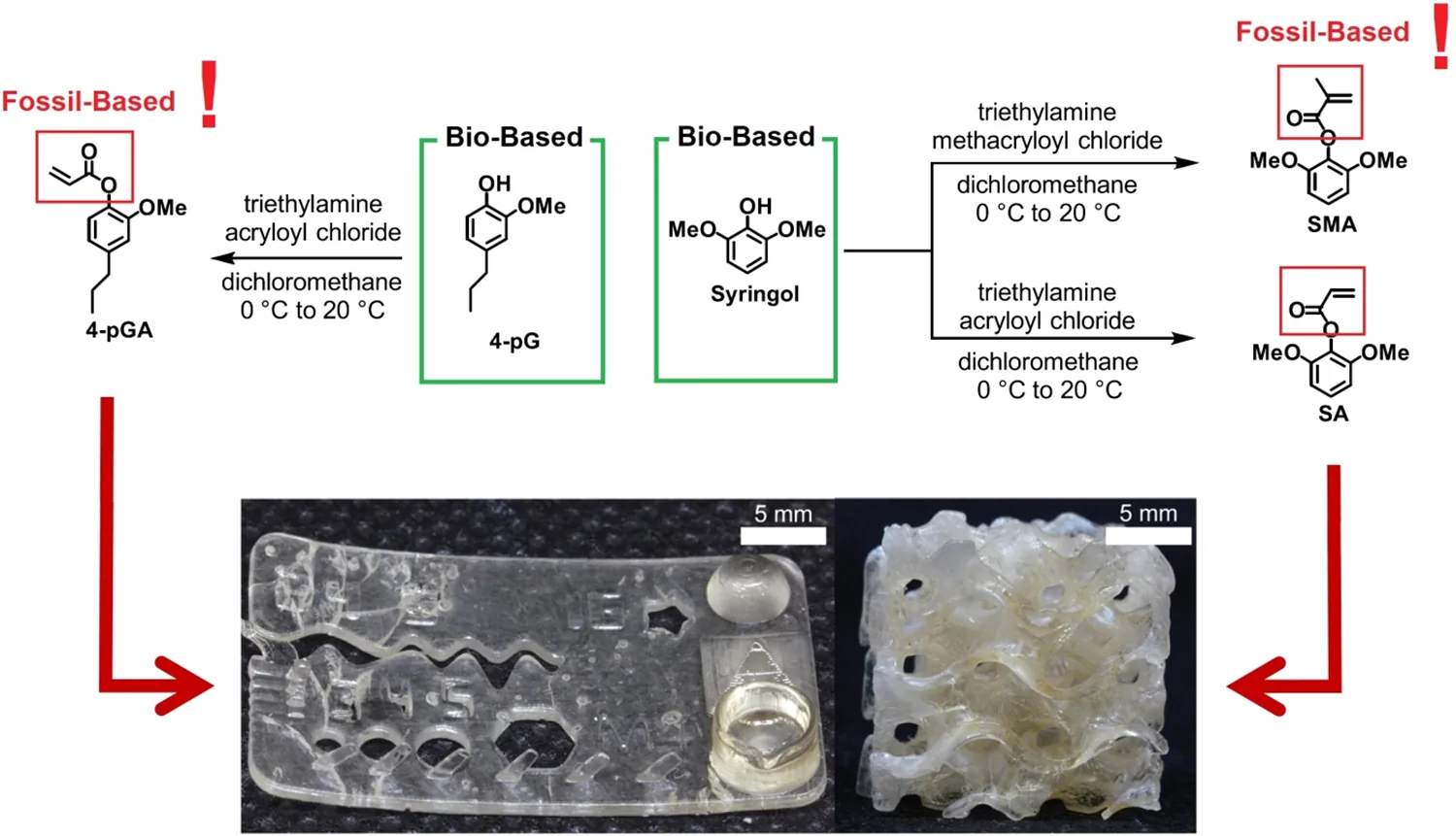
Partly fossil-based precursors for vat photopolymerization derived from biobased lignin derivatives—4-propylguaiacol, syringol, and modified by acrylate and methacrylate polymerizable functional groups.^93^ Adapted with permission from ref 93. Copyright 2023 American Chemical Society.

**Figure 3:**
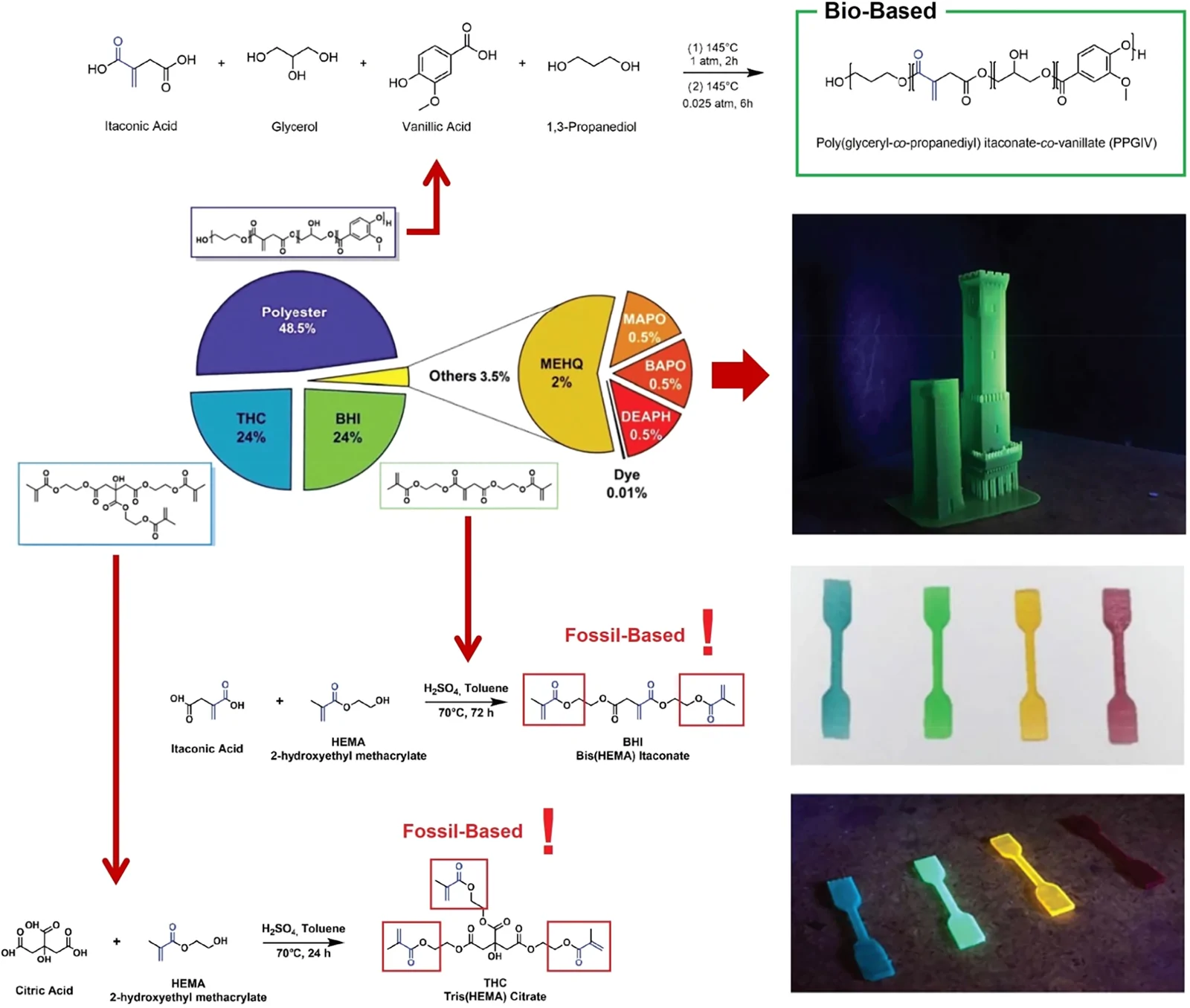
Combination of a fully biobased polyester structure derived from itaconic acid, glycerol, vanillic acid, and 1,3-propanediol, enhanced by the partly fossil-based reactive diluent—bis­(HEMA) itaconate (BHI), and the partly fossil-based cross-linker—tris­(HEMA) citrate (THC).^94^ Adapted with permission under a Creative Commons CC-BY-NC-3.0 from ref 94. Copyright 2020 Royal Society of Chemistry.

Selected published investigations reported entirely biobased, suitable systems for vat photopolymerization, engineered through a polyester structure approach or a bioderived epoxy system (see Figure 4). Maturi et al.^96^ reported an interesting synthetic approach to a fully biobased polyester system, incorporating three reaction steps to yield tough, flexible systems for vat photopolymerization. First, the authors used biotechnologically derived itaconate dimethyl ester in a Diels–Alder cycloaddition reaction with the natural terpene myrcene. This approach leads to a cycloadduct containing esterified methanol structures capable of bulk condensation via transesterification with additional dimethyl itaconate and a selected diol, forming a photocurable, highly viscous polyester. Eventually, this highly viscous system was modified with various monomer structures, yielding tough, flexible, vat photopolymerized thermosets. While the tough polymers were completely biobased, using 1,4-butanediyl bis­(methyl itaconate) and isopropyl (4-hydroxybutyl) itaconate, the flexible thermoset incorporated methacrylate derivatives, particularly 2-(methacryloyloxy)­ethyl dodecanoate and bis 2-(methacryloyloxy)­ethyl dodecanedioate.

**Figure 4:**
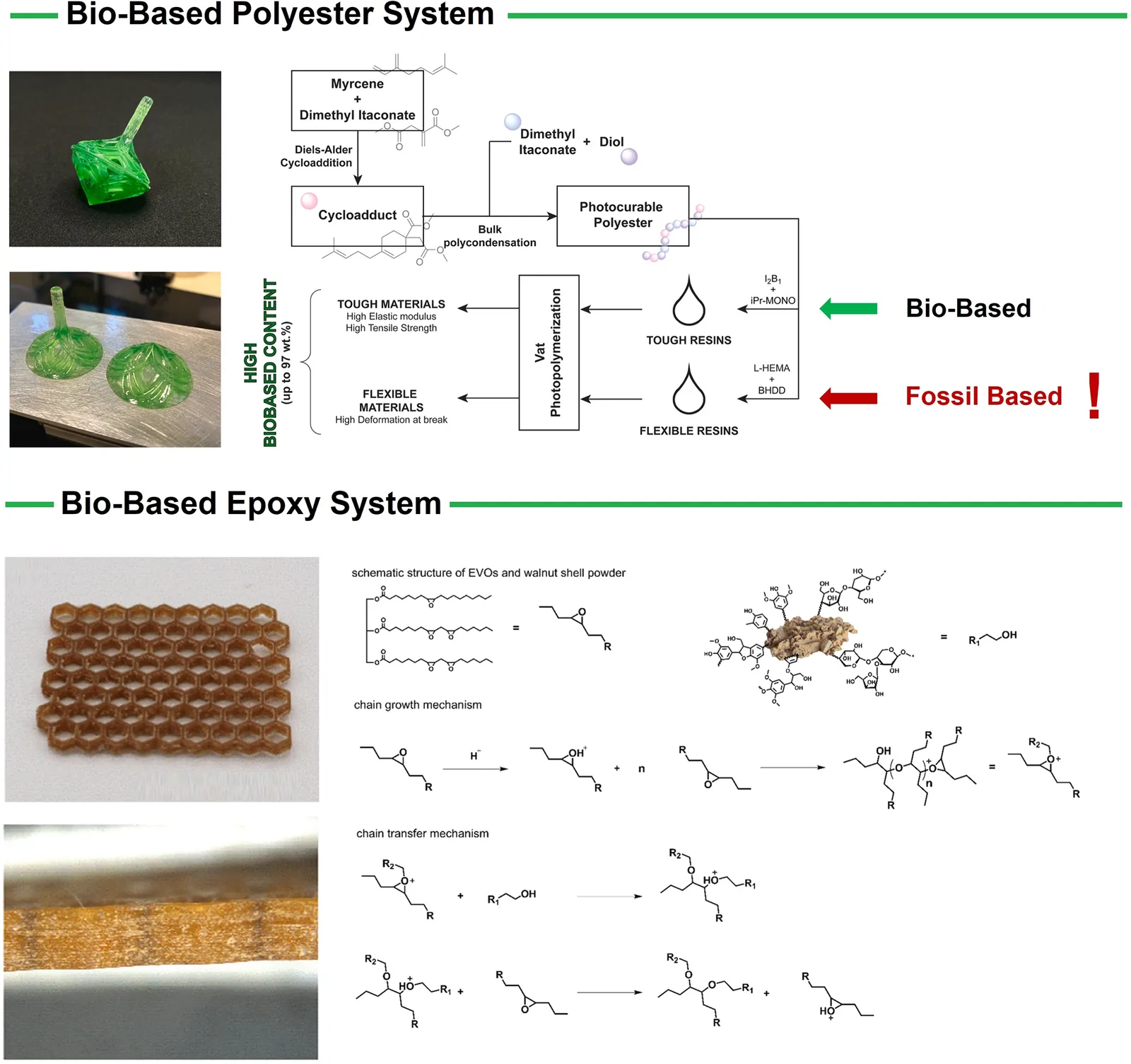
Approaches to fully biobased 3D-printing resins. Top: fully biobased polyester resin derived from itaconic acid ester and terpene myrcene. Bottom: fully biobased epoxy resin based on epoxidized linseed oil (ELO) or epoxidized soybean oil (ESO) and bioderived fillers from walnut shells and hemp.^96,97^ Reproduced from ref 96 under a Creative Commons CC-BY-4.0. Copyright 2024 Elsevier. Reproduced from ref 97 under a Creative Commons CC-BY-4.0. Copyright 2023 Elsevier.

Similar to the previously mentioned investigations, the biobased polyester structures require several rheological modifications, incorporating fully biobased monomers in the described experimental research. Another investigation proposed fully biobased 3D-printable systems based on the epoxidized linseed oil (ELO) and epoxidized soybean oil (ESO).^97^ Since commonly epoxidized oils are solid phase at room temperature, the authors applied the Hot Lithography process to achieve additive manufacturing using epoxidized triacylglycerides. The optimal temperature for the ELO gel time of 4.5 s was 100 °C. Also, the authors employed bioderived fillers to enhance the mechanical performance of the 3D-printed systems, using walnut shells and hemp. The combination of the fully biobased reactive precursor with the bioderived fillers achieved a tensile strength of 10 MPa, comparable to that of acrylate-based resins (according to the authors). It should be noted that although the epoxy-based oil system was processed by hot lithography, several specific epoxidized biobased systems remain liquid at room temperature and offer many advantages for sustainable vat photopolymerization. In particular, acrylated and methacrylated oils and systems are oxygen-inhibited in air, leaving a sticky surface on 3D-printed objects. This phenomenon is caused by the formation of peroxy radicals, which inhibit propagation. Deeper in the cured resin, oxygen is no longer present; therefore, the object can be polymerized throughout, except for the surface. Epoxy systems cured through cationic polymerization do not suffer this obstacle, which favors them for vat photopolymerization. However, cationic polymerization initiators must be considered, even though their hazards differ from those of radical initiators. Eventually, epoxidized precursors are often prepared directly by modifying reactive epoxy intermediates; therefore, the straightforward application of epoxy resins reduces the need for additional structural modifications.

Itaconic acid-derived compounds and systems belong among the most promising representatives for biobased vat photopolymerization. Several recent investigations have focused on and described various systems incorporating itaconic acid and/or its numerous derivatives. Spanu et al.^98^ demonstrated a perspective synthesis of a photocurable main resin derived from sorbitol- and caprolactone-itaconic derivative, entitled sorbitol hexa­(pentacaprolactone methyl itaconate). In addition to the production of a highly viscous main resin, several biobased reactive diluents were also employed, namely, dodecyl methyl itaconate, 1,4-butanediyl mono­(methyl itaconate), and 1,4-butanediyl bis­(methyl itaconate). These engineered biobased complex systems were mixed with several fossil-based compounds (pentaerythritol triacrylate, glycerol dimethacrylate, and glycerol propoxylate triacrylate), indicating the need for additional supporting molecules. The authors demonstrated a successful 3D printing process; however, cross-linkers from petroleum-based sources were required. An alternative strategy was demonstrated by another group, which synthesized an oligoester structure from polycaprolactone (PCL), 1,4-butanediol, and itaconic acid to obtain low-*T*_g_ biobased resin precursors.^99^ As in the first-mentioned article, the successful 3D printing of the synthesized itaconic acid ester was demonstrated. Polyester itaconic acid structures enhanced with compounds containing disulfide linkages ensure the potential recyclability of the 3D-printing thermoset resins, a highly demanded strategy. Papadopoulos et al.^100^ synthesized photocurable polyesters from itaconic and sebacic acids, 1,3-propanediol, and 1,12-dodecanediol, enhanced with 3,3′-dithiodipropionic acid, which provides dynamic covalent bonding. The disulfide bonds can be debonded and recycled at higher temperature and pressure (in this case at 160 °C and 20 MPa after 1 h). Although the original polyester resin loses a considerable portion of its mechanical performance (20–40% of its original values), the 3D-printed products do not necessarily need to exhibit superior limit strength and exceptional rigidity for suitable applications.

Besides ester derivatives of itaconic acid, which exemplify the most straightforward potential candidate for vat photopolymerization as a main resin, several other reactive systems have been synthesized and applied for additive manufacturing in the published literature. Carmenini et al.^101^ incorporated monomethyl itaconoyl chloride into the structure of biobased glycerol carbonate. Acyl halides exemplify the most reactive acyl donors for the production of photocurable precursors. The authors applied the produced glycerol carbonate methyl itaconate to soft photocurable resins comprising ethylene glycol phenyl ether methacrylate and 1,6-hexanediol diacrylate and to rigid thermosets containing glycerol dimethacrylate and glycerol propoxylate triacrylate. Again, the authors decided to formulate the final printable resin from itaconic acid derivatives, enhanced with fossil-based, high-performance additives to ensure efficient vat photopolymerization. Aside from carbonate-itaconate derivatives, other published investigations have focused on isocyanate-free urethane diol itaconates, which combine carbamate and ester bonds in the carbon backbone, resulting in unique and enhanced mechanical properties compared to simple oligoesters. The mechanical characterization (tensile and flexural) confirmed that the itaconic-urethane structure exhibited competitive performance to the acrylate/methacrylate-based glycerol dimethacrylate and glycerol propoxylate triacrylate. Although the curing times were set much higher than those used in commercial vat photopolymerization (80–120 s/layer), the resulting thermoset exhibited exceptional material performance.

Next to the acyl halides used as structure modifiers, itaconic anhydride plays a crucial role in the production of biobased resins for vat photopolymerization. Chiaradia et al.^102^ demonstrated an alternative esterification reaction, the Fisher esterification, using itaconic anhydride to modify perillyl alcohol, which is a naturally occurring monoterpene. Unlike previous synthesized esters cured via radical polymerization, the authors employed a thiol–ene click curing mechanism in their polymerizable resins. In addition to the synthesis of the main precursor, the use of supporting reactive diluents is efficient, as it allows the use of unmodified biobased compounds. Molecules such as limonene or linalool contain unsaturated double bonds, which are incorporated into the thiol–ene polymerized structure, while the sole terpene compounds remain completely chemically unmodified. This approach represents a highly prospective strategy, in terms of production simplicity and potential environmental safety. Apart from the modification of free hydroxyl groups, amide structures derived from itaconic acid represent innovative, structurally promising curable precursors.^103^ The researchers produced five different amide-itaconic compounds serving as main resin precursors (see Figure 5) diluted by selected biobased additives, isobornyl methacrylate, ethylene glycol phenyl ether acrylate, and hexynediol diacrylate. The authors demonstrated the successful synthesis of all declared products, achieved efficient, high-resolution 3D printing, and observed mechanical properties comparable to those of commercial precursor alternatives.

**Figure 5:**
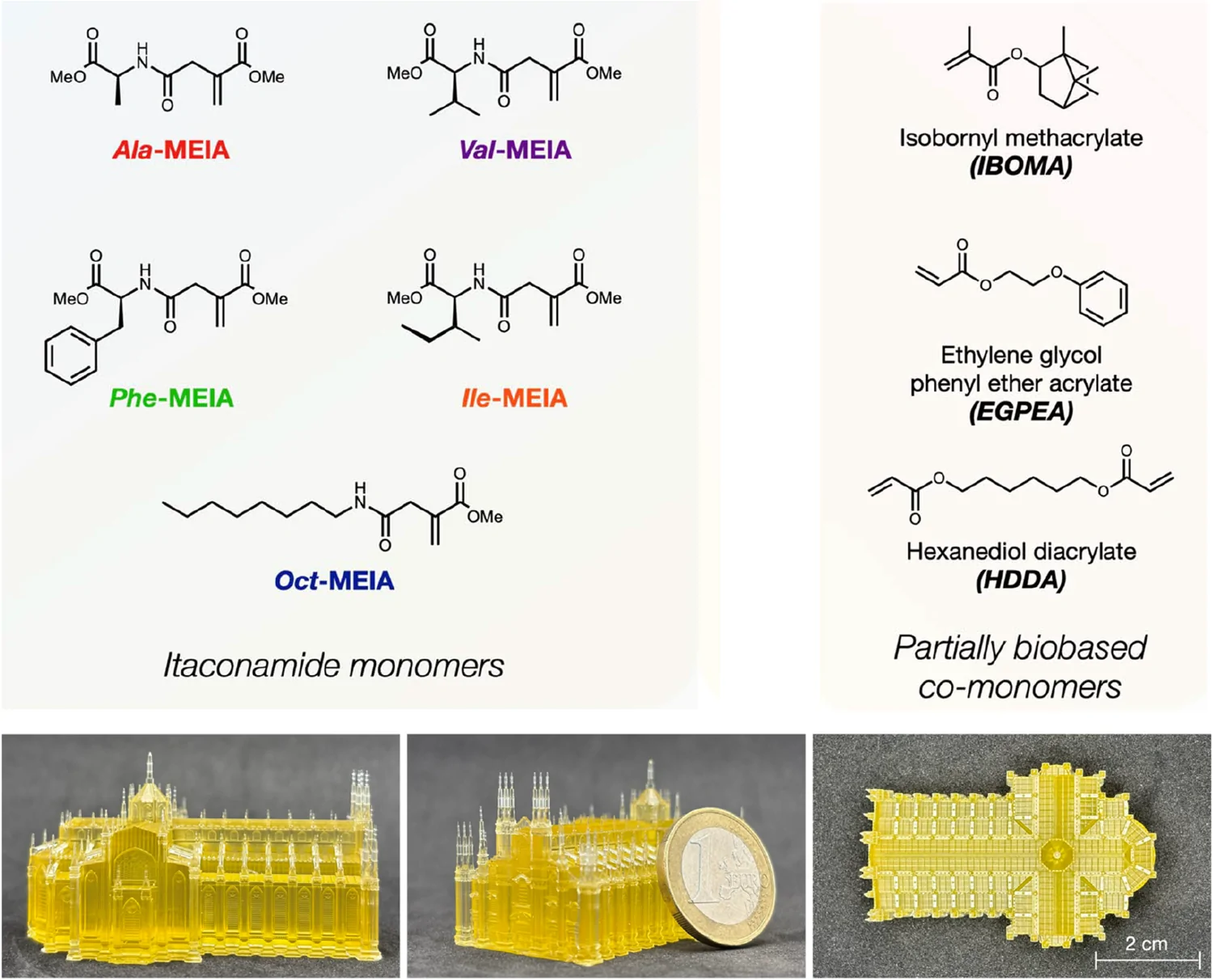
Five different synthesized itaconamide monomers produced by nucleophilic substitution of selected amino acids and itaconic anhydride. The main itaconamide resin was diluted with isobornyl methacrylate, ethylene glycol phenyl ether acrylate, and hexanediol diacrylate to form a suitable precursor mixture for vat photopolymerization. Reproduced from ref 103 under a Creative Commons license CC-BY-NC-4.0. Copyright 2026 Royal Society of Chemistry.

The polyester structures tailored from various biobased and petroleum-based compounds often exhibit issues with optimal flow properties, similar to those of modified vegetable oils. Their main advantage lies in the detailed molecular engineering offered by the various structural compositions of the selected components in the polyester. Either very rigid or elastic reactive precursors can be suggested and synthesized. The synthesis is complicated by the reaction conditions (high temperatures, 100–200 °C, and reduced pressure to remove water formed during esterification). Compared to the modification of vegetable oils for 3D printing, esterification approaches entail a complex production process. The overall reactivity of 3D-printable precursors based on fossil-based oils and engineered polyesters represents the most significant advantage over the reported fully biobased compounds composed of epoxidized structures^104^ and radically polymerizable itaconates.^105^ Apart from the increased reactivity, which extends 3D printing time, the fully biobased systems hold enormous potential for commercial vat photopolymerization due to their health and safety, negligible volatility, and lower environmental impact.

The biobased alternatives exhibit considerable limitations compared to the fossil-based, high-performing photocurable alternatives based on acrylates or methacrylates. Table 1 summarizes the most important properties relevant to vat photopolymerization that directly support successful 3D printing. The main advantages and disadvantages of fully biobased resins are illustrated in Figure 6.

**Figure 6:**
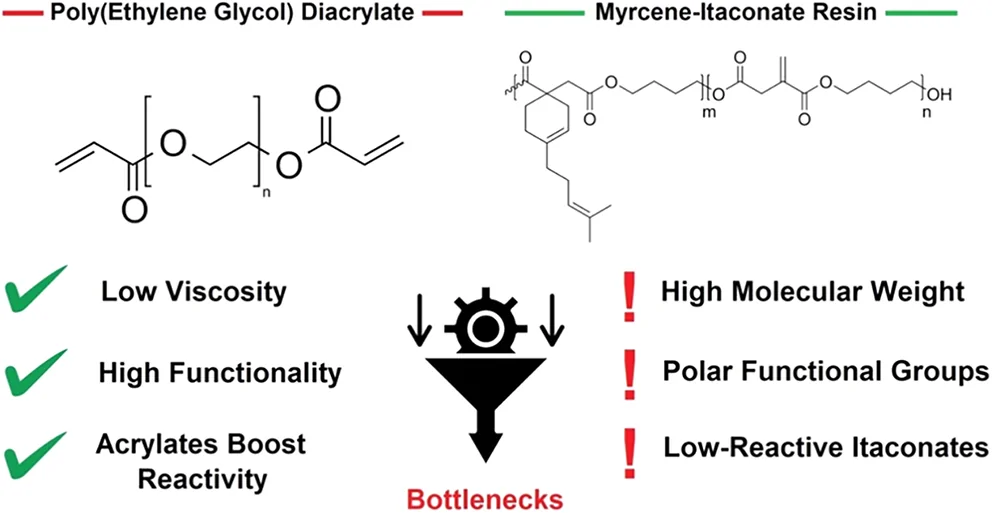
Main functional advantages of the fossil-based photocurable resins (represented by poly­(ethylene glycol) diacrylate) and the challenging bottlenecks of the biobased reactive systems (represented by myrcene-itaconic resin).

**Table 1:** Comparison of Critical Material Properties of the Selected Photocurable Resins for the Vat Photopolymerization Process

system	viscosity	reactivity	tensile strength	biobased content	reference
acrylated epoxidized soybean oil	10,000–15,000 mPa·s at 25 °C	critical energy of 1.44 mJ·mm^–2^	below 10 MPa	78.5–84.5%	^88^
polyester with bis(HEMA) itaconate and tris(HEMA) citrate	100–200 mPa·s at 25 °C	10 s of exposure time/layer	5.4 ± 0.7 MPa	around 96.5%	^94^
isosobride and itaconic acid polyester	91,780 mPa·s at 23 °C	conversion of 50% after 55 s	unable to Print	95%	^95^
polyester from acrylic acid and fossil-based C6 polyols (3D-printed reference)	2310 mPa·s at 23 °C	conversion of 100% after 5 s	0.82 ± 0.07 MPa	0%	^95^
myrcene-itaconate biobased resin produced via diels–alder	589–29,200 mPa·s at 25 °C	100 s irradiation time to cure required	1.76–15.6 MPa	79.5–97.0%	^96^
epoxidized linseed oil for hot lithography	800–1300 mPa·s at 25 °C	conversion of 65% after 300 s at 100 °C	around 1.0 MPa	97.0%	^97^
epoxidized soybean oil for hot lithography	400–600 mPa·s at 25 °C	conversion of 45% after 300 s at 100 °C	around 11.0 MPa	97.0%	^97^
itaconic acid-functionalized polycaprolactone	340–3100 mPa·s at 25 °C	100 s of exposure time per layer	3.6–12.1 MPa	29.0–97.0%	^98^
polycaprolactone-itaconic acid resin	around 100 mPa·s at 25 °C	22 s exposure time/layer	2.4–5.2 MPa	23.5–27.5%	^99^
itaconic acid recyclable polyester resin	below 5000 mPa·s at 25 °C	5–10 s exposure time/layer	1.9–4.3 MPa	15.7–87.6%	^100^
glycerol carbonate methyl itaconate	10–100 mPa·s at 27 °C	80–120 s exposure time/layer	9.2–41.7 MPa	77%	^101^
terpene-itanonate anhydride-based resin	60–120 mPa·s at RT	110 s exposure time/layer	12.5–32–5 MPa	36.6–63.6%	^102^
itaconamine monomers	below 5000 mPa·s at 25 °C	50–120 s of exposure time per layer	17.2–40.2 MPa	43.5–70.4%	^103^

As listed in this section, many chemically distinguished compounds are synthesized from various biobased sources to serve as the main resin for vat photopolymerization, with the curing mechanism being the main factor significantly affecting the eventual efficiency and applicability of the engineering systems. Most of the references and discussed systems rely on the radical polymerization mechanism using specific photoinitiators among the wide variety of available compounds. This approach ensures fast curing, straightforward preparation, and high polymerization conversion. On the other hand, commercial photoinitiators are usually toxic and hazardous. In addition to radical polymerization, cationic polymerization is often employed for epoxy-based systems. However, the required initiators for such a strategy are also significantly hazardous. The thiol–ene polymerization usually requires irradiation at lower wavelengths (starting with UV-A), which, in theory, represents a more harmful approach. On the other hand, such systems require minimal chemical modification as unsaturated biobased compounds are available and affordable. The combination of an initiatorless approach with suitable process conditions (sufficient protection of vat photopolymerization users and photocurable resins against UV irradiation) may ensure a safer, more environmentally friendly approach in additive manufacturing.

## 3 Reactive Diluents

Vat photopolymerization depends heavily on the precise engineering of the rheological profile of the curable reactive precursor used, given its functional purpose—excessively viscous systems are problematic due to suboptimal 3D-printing platform operation, as evidenced by excessive wetting and the polymerizable system’s affinity for the moving 3D printer platform.^106,107^ Several published investigations reported that the limit viscosity of efficient and optimal additive manufacturing through vat photopolymerization should not exceed 5000 mPa·s.^108–110^ As described and discussed earlier, most partially and fully biobased systems, such as specific polyesters, epoxy compounds, and terpene derivatives, exhibit extremely high viscosities, reaching tens of Pa·s.^111,112^ Besides the reported myrcene as a prospective terpene, several other biobased compounds represent modifiable options optimal for reactive systems. Limonene contains two reactive alkene groups that can be polymerized to form various biobased resins, as reported in the literature.^113^ Thiol–ene reactions require unsaturated alkenes and thiol (R–SH) for a photoinitiated reaction that does not need further photoinitiation. This approach may be prospective since photoinitiators can be avoided.^114^ Geraniol represents a potential compound for biobased vat photopolymerization,^115^ together with carvone reactive derivatives.^116^

Due to the aforementioned aspects, specific assisting compounds, reported as reactive diluents, are usually used to modify the flow properties of reactive systems, thereby transforming them into suitable precursors for vat photopolymerization. Reactive diluents are primarily used to reduce excessively high viscosity, providing optimal printing characteristics for the systems they are used with.^117^ Moreover, since such compounds can comprise many different functional groups and bonded structures, such as esters, amides, ethers, aromatic structures, free hydroxyls, sulfides, and other structural components,^117^ reactive diluents also affect the overall mechanical and thermo-mechanical performance,^118^ modify the eventual surface energies and precursor’s surface tension,^119^ improve the thermal stability of the 3D-printed resins,^120^ enhance the overall photoreactivity of the processed system,^121^ or provide unique final properties, such as pH-responsive character^122^ or vitrimer behavior.^123^

As discussed earlier, vat photopolymerization primarily uses radically polymerizable acrylates and methacrylates, as well as cationically polymerizable epoxy compounds, for optimal processing. Both of these structural reactive compound types also form low-molecular-weight monomer structures, which are commonly used as reactive diluents.^124^ Most commonly used systems, such as styrene or methyl methacrylate, are industrially used for high-scale material fabrication.^125^ However, these compounds are highly volatile, which prohibits their successful application in vat photopolymerization.^126^ This technology ideally requires practically nonvolatile systems, as it operates in open-vat conditions. More suitable alternatives derived from petroleum sources include ethylene glycol diacrylate (EGDA), ethylene glycol dimethacrylate (EGDMA), diethylene glycol diacrylate (DEGDA), and others.^117^ Cationic polymerized systems for additive manufacturing also naturally require epoxy-based reactive diluents for optimal operation. Several monofunctional fossil-based compounds with exceptional diluting properties are commercially used, namely, phenyl glycidyl ether (PGE) and 2-ethylhexyl glycidyl ether (EHGE).^127,128^ Besides ensuring lower viscosity levels, numerous di- and multifunctional epoxy systems are applied for the enhancement of the eventual thermoset’s properties, such as 1,4-butanediol/neopentyl/diethylene glycol diglycidyl ether (BDDE, NPDGE, DEGDE),^129–131^ trimethylolpropane triglycidyl ether (TMPTGE),^132^ or glycerol triglycidyl ether (GTE).^133^ All of the listed reactive compounds are entirely fossil-based, which represents an unsustainable approach for vat photopolymerization and poses countless potential health hazards and environmental risks.

Bioinspired reactive diluents represent an alternative to the commercially used systems listed above, offering increased sustainability, incorporating biobased starting materials, and potentially mitigating health and environmental risks.^117^ The available literature reports several partly biobased molecular systems that incorporate fossil-based functional modifications, similar to the previously listed acrylated or methacrylated main photocurable resins (see Figure 7).^134,135^ Even though this approach does not exclusively use biobased starting substances, the resulting alternatives at least contain partly biobased compounds. Suitable partly biobased systems for radically polymerized precursors reported in the literature are isobornyl acrylate/methacrylate (IBOA/IBOMA),^136,137^ vanillin methacrylate (VMA),^138^ cinnamyl methacrylate (CMA),^139^ or glycerol trimethacrylate (GTMA).^140^ Additionally, 2,5-bis­(hydroxymethyl)­furan is a potential starting material for several engineered reactive diluents with diverse properties.^141^ The Achilles’ heel of the listed partly biobased systems is their synthesis and processing. Such approaches use highly available and biobased starting materials and substances. However, several fossil-based and hazardous modifying molecules must be employed for the successful preparation of reactive diluents, namely, acryloyl and methacryloyl halides,^144^ methacrylic anhydride,^142^ acrylic and methacrylic acid and their esters,^143,144^ along with several unsustainable catalysts, such as 4-dimethylaminopyridine (DMAP)^145^ or triethylamine (TEA),^146^ and supporting chlorinated and petroleum-based solvents, namely, dichloromethane,^147^ chloroform,^148^ toluene,^149^ or tetrahydrofuran.^150^ The incorporation of the acrylate/methacrylate-based reactant, along with the hazardous catalysts and solvents, decreases overall sustainability. Low-molecular-weight molecules containing epoxy functional groups could represent a suitable, fully biobased alternative to the mentioned acrylates and methacrylates. However, most of the investigated epoxy reactive diluents use a highly unsustainable epoxidizing agent, 3-chloroperbenzoic acid (MCPBA).^151,152^ This compound is widely used for the epoxidization of unsaturated double bonds within several biobased substances, such as cardanol,^151^ fatty acids,^153^ limonene,^154^ or pinene.^155^ Hydrogen peroxide-based epoxidization mixtures exemplify an alternative synthetic approach toward fully biobased epoxy compounds for reactive dilution; however, the optimal catalysis remains a serious obstacle to this strategy.^156^

**Figure 7:**
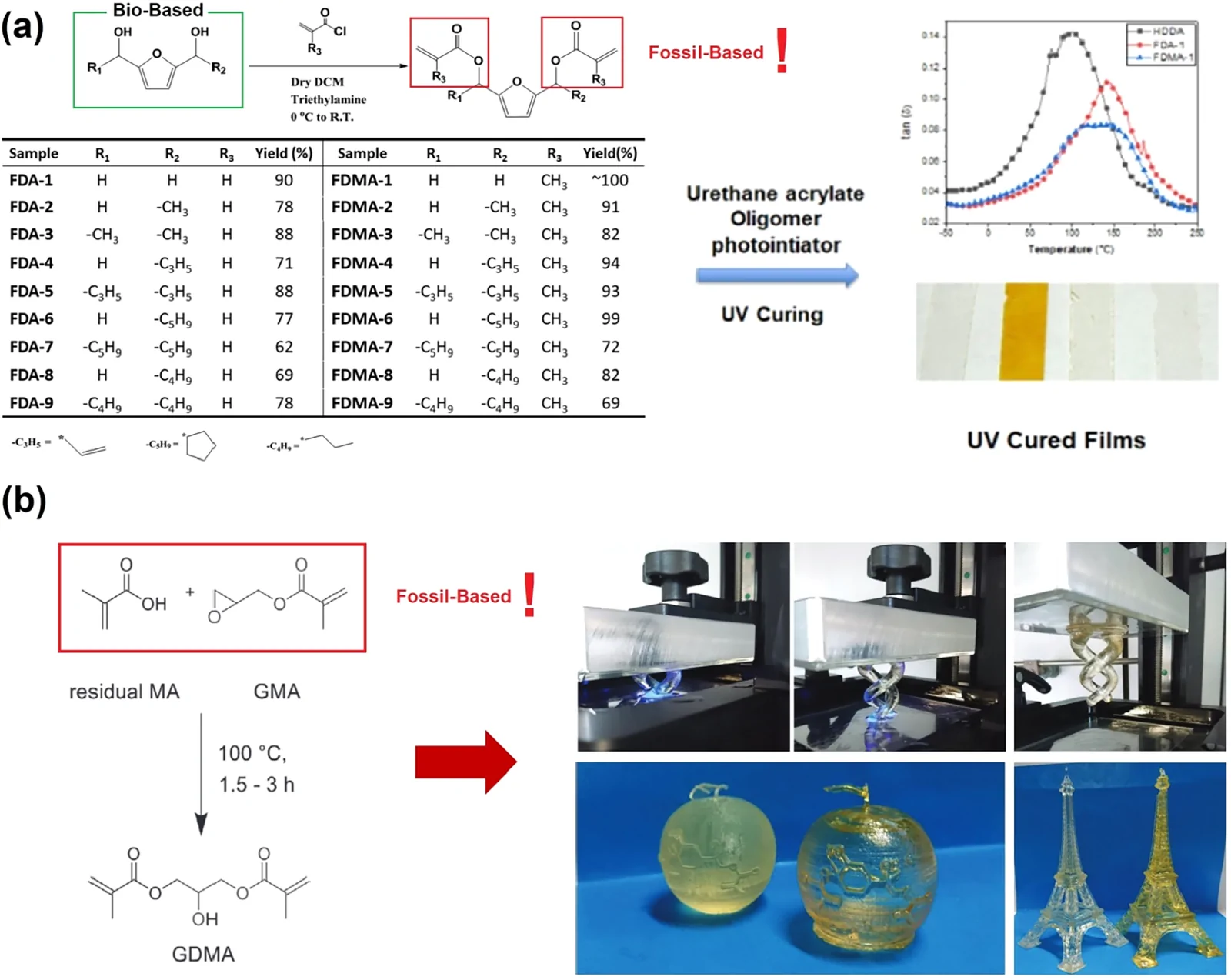
(a) The partly biobased reactive diluents derived from 2,5-bis­(hydroxymethyl)­furan for reactive diluting of highly viscous urethane acrylate oligomers used for UV-cured films.^134^ Adapted with permission from ref 134. Copyright 2021 American Chemical Society. (b) Synthesized glycerol dimethacrylate entirely from fossil-based starting substances after the synthesis of curable limonene-based 3D-printing resin.^135^ Reproduced from ref 135 under a Creative Commons CC-BY-4.0. Copyright 2020 WILEY-VCH Verlag GmbH & Co. KGaA.

Fully biobased reactive diluents are currently attracting significant attention due to their potential to reduce environmental and health risks. Most of the described, debated, and investigated systems are synthesized from itaconic acid, a C5 dicarboxylic acid that possesses an alkene capable of radical polymerization (Figure 8).^157^ This starting molecule is produced biotechnologically by several microbial producers, including *Aspergillus terreus*, *Ustilago maydis*, and *Pseudozyma tsukubaensis*.^158,159^ Since this compound contains two reactive carboxyl functional groups, it can undergo several reactions, such as esterification, cyclic dehydration, or amidation.^157,160^ In the fabrication of reactive diluents, esterification and dehydration (to produce itaconic acid anhydride) play a key role due to the products’ physicochemical properties.^161^ Suitable mono- and diesters of itaconic acid exhibit low viscosity, which is essential for reactive diluting. The most recent published works report a comprehensive investigation focused on the application and description of mono- and diesters of itaconic acid, summarizing the diluting properties, mechanical performance, and also the overall reactivity of the compounds, which is affected by the functional groups in the system (polar functional groups, such as free hydroxyls, enhance the overall reactivity toward radical polymerization).^161^ Regarding the ester compounds, Arnaud et al.^162^ published an investigation dedicated to heterodiesters, which are itaconates containing two different esterified alcohols in their structure. This approach significantly affects the overall properties and performance. The authors also processed their heterogeneous itaconates via photocuring. Additionally, another investigation by the same main author focused on the role of synthesized itaconic anhydride in the production of heteroesters.^163^ This strategy ensures a more convenient formation of monoesters involving highly volatile alcohols, such as methanol or ethanol, due to the anhydride’s reactivity at lower temperatures compared to Fischer esterification. An exception to the commonly discussed itaconates for reactive diluting exemplifies 4-vinylguaiacol acetyl ester (AC4VG), produced entirely from biobased sources by Dai and the team (see Figure 8).^164^ The authors transformed ferulic acid into a polymerizable and low-viscosity AC4VG via a two-step synthesis, and they verified the diluting properties by modifying the working viscosity of their prepared highly viscous polyester precursor.

**Figure 8:**
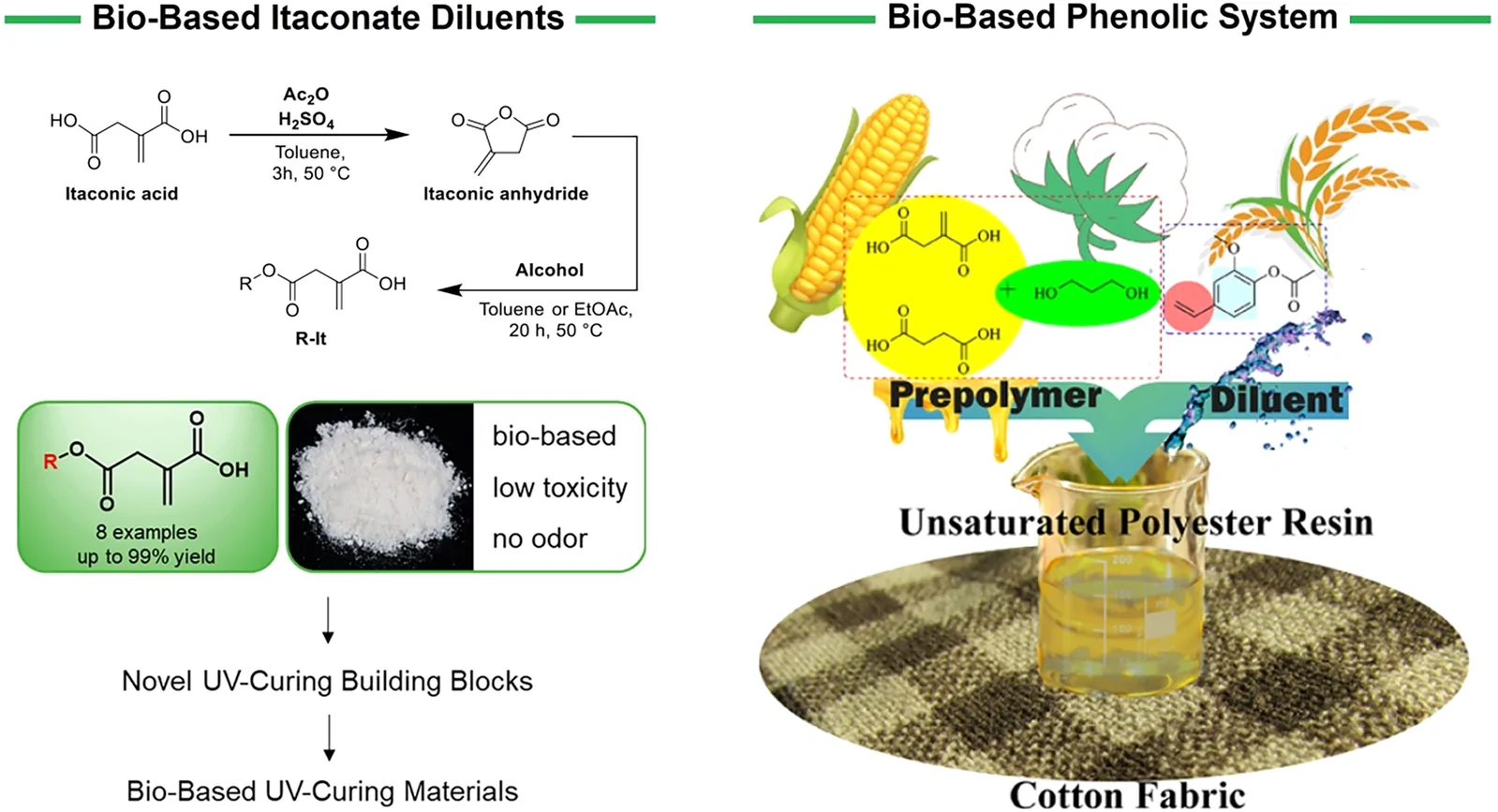
Two different published approaches toward fully biobased reactive diluents for (photo)­curing applications based on itaconic acid derivatives^163^ and modified ferulic acid.^164^ Adapted with permission from ref 163, copyright 2019 American Chemical Society, and from ref 164, copyright 2020 American Chemical Society.

Based on the described chemical structures of the main photocurable resins and reactive diluents, we summarized key carbon backbone functionalities and factors to consider in the engineering of biobased systems suitable for vat photopolymerization. The listed parameters include key functional characteristics, photocuring reactivity, and the specific mechanical performance of the 3D-printed thermoset resins. The advice is listed below:

- **Optimal flow characteristics:** Due to the polyester, generally oligomer, or branched triacylglyceride structure of the reported biobased substances, the viscosity is strongly recommended to be modified via suitable reactive diluents. Based on the summarized findings, only hot lithography may overcome the high viscosity issue on the majority of the partially or fully biobased precursors. Generally, polar and hydrogen-bond-forming functional groups contribute significantly to the high viscosity (hydroxyls, amines, amides, urethanes). Such functional groups should be minimized, especially in the main photocurable resins of polymers and oligomers.
- **Photocuring reactivity:** The optimal structural composition of the reactive components in 3D printing precursors is essential. The main photocurable resin should contain multiple polymerizable functional groups. Reactivity enhancement can be ensured by selecting an optimal reactive diluent. Generally, optimal low-molecular-weight chemical structures increase photoreactivity. Additionally, polar functional groups increase polymerization activity but should be localized in the reactive diluents to avoid increasing the precursor’s overall viscosity.
- **Mechanical performance:** Suitable long-chain biobased compounds must be selected to achieve flexible 3D-printed resins. On the other hand, the increase in cross-linking density, together with rigid, ideally planar chemical structures, results in highly strong photocured resins.

We summarized the critical properties of the reported biobased reactive diluents relative to fossil-derived alternatives in Table 2 to illustrate the key differences comprehensively. The main challenges for the fully biobased reactive diluents are illustrated in Figure 9.

**Figure 9:**
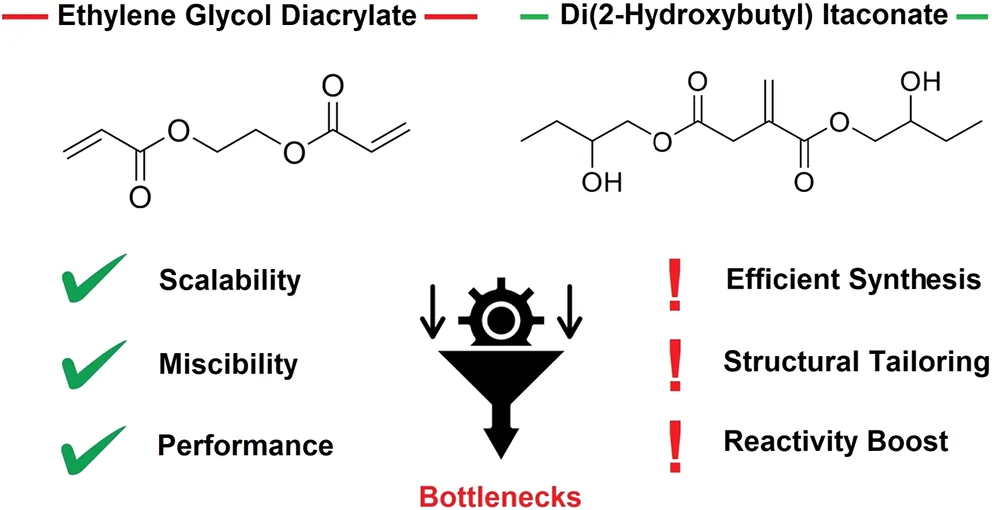
Main functional advantages of the fossil-based reactive diluents (represented by ethylene glycol diacrylate), and the challenging bottlenecks of the biobased reactive diluents (represented by di­(2-hydroxybutyl) itaconate).

**Table 2:** Comparison of the Functional Properties of the Selected Reactive Diluents

reactive diluent	viscosity	reactivity	reference
isosorbide acrylate	decrease from 1000 Pa·s to 1.5 Pa·s (50 wt % of diluent in commercial resin) at 25 °C	above 83% conversion after 800 s irradiation time	^179^
isosorbide methacrylate	decrease from 1000 Pa·s to 0.7 Pa·s (50 wt % of diluent in commercial resin) at 25 °C	above 68% conversion after 800 s irradiation time	^179^
triethylene glycol dimethacrylate (TEGDMA)	9 mPa·s at 25 °C	79 *±* 2 conversion after 200 s irradiation time	^180^
dibutyl itaconate	7 mPa·s at 25 °C	conversion of 30% after 570 s	^162^
dicyclohexyl itaconate	103 mPa·s at 25 °C	conversion of 58% after 570 s	^162^
di(2-hydroxybutyl) itaconate	968 mPa·s at 25 °C	conversion of 65% after 570 s	^162^
4-vinylguaiacol acetyl ester (AC4VG)	2.89 mPa·s at 50 °C	not investigated	^164^

Sufficient reactivity was mentioned several times throughout this perspective. Mostly, the polymerization activity was evaluated using universal approaches, as selective investigation of photocuring was performed before vat photopolymerization.^162,164,179,180^ To evaluate photocuring reactivity and polymerization rate, photo-DSC^183^ and real-time FT-IR^184^ methods are commonly used due to their universal applicability and low material consumption. In the context of vat photopolymerization, Jacob’s working curve is commonly used to determine optimal 3D-printing parameters for a specific resin type. The equation stands as follows^185^

1 $$C_{d}=D_{p}\cdot \ln\left(\frac{I_{0}t}{E_{c}}\right)$$

where *C*_d_ is the curing depth (m), the slope (*D*_p_) exemplifies the penetration depth (m), *I*_0_ stands for the irradiation source intensity (W/m^2^), *t* is the exposure time (s), and *E*_c_ is the critical curing energy (J/m^2^). The possible limitations of fully biobased resins and reactive diluents lie primarily in their lower reactivity compared to commercial, highly reactive alternatives. However, the working curve was previously successfully used with itaconate derivatives in 3D printing.^186^ The primary negative aspect of using Jacob’s working curve is the amount of resin required and the potential for high viscosity. Since the curve uses 3D-printed layer thicknesses fabricated by a 3D printer, the flow characteristics might impede the efficient performance of such a process, along with the incomparably higher amount of working resin. This approach is suitable for ideally low-toxic, volatile systems, which can be produced in large quantities (hundreds of grams) to provide sufficient quantity and safety during the reactivity investigation.

Economic factors play the most significant role in scalability, applicability, and competitiveness when comparing fully biobased compounds and systems to commercially used precursors. Itaconic acid represents one of the linking biobased compounds suitable for the economic comparisons—the current price is stabilized at 1.5–2.0 USD/kg.^181^ The stabilized price of acrylic acid (reported in 2021) reached 1.40–1.65 USD/kg, which is not significantly lower than that of itaconic acid.^182^ Moreover, several functional derivatives of both acrylic and methacrylic acid are commonly used in the available literature (acyl halides and anhydrides), which represent even more expensive entering compounds. On the other hand, itaconic acid usually requires high-temperature Fischer esterification,^94^ or multistep continuous chemical syntheses to form perspective compounds.^162,163^ The scalability and expanding applicability are directly dependent on the prospective synthesis strategy, which connects a straightforward approach with minimal energetically demanding processes. The polyester structures synthesized from itaconic acid are expected to consume substantial energy due to the reaction’s nature. However, reactive diluents based on itaconates can be efficiently synthesized from readily available compounds using a specifically engineered process (cyclic anhydride opening or azeotropic distillation).

Regarding reactive diluents employed in vat photopolymerization systems, the rheological modifications, the achieved polymerization activity, and the minimization of health and environmental hazards are key aspects for the prospective enhancement of additive manufacturing technologies. The optimal miscibility and chemical structure compatibility between the main resin and the employed reactive diluents result in efficient rheological modification and a suitable functional character of the prepared photopolymerization precursor. Typically, molecules with similar functional groups and solubility parameters form efficient systems (e.g., the fabrication of ester main resins with esterified low-molecular diluents, or combining strictly nonpolar structures lacking free hydroxyl or amine/amide groups with similarly hydrophobic diluents is a prospective strategy). Aside from miscibility and rheological optimization, curing activity is a significant parameter in the context of vat photopolymerization applications (current commercial systems form thin layers (50 μm) in a matter of seconds). The lower the reactive diluent’s absorption of irradiation, the higher the polymerization rate due to more efficient initiation by traditional photoinitiators (does not apply to thiol–ene systems). Therefore, the absorption profile of the reactive diluent and also the main resin should be monitored and regulated. In addition to its irradiation-absorbing properties, the molecular weight of the reactive diluent plays a vital role in overall reactivity. Low-molecular, highly functional compounds typically reach faster polymerization. All of the listed factors should be considered in the context of the vat photopolymerization application.

## 4 Biobased Photoinitiators

Photoinitiators are an indispensable part of 3D-printing resins, whether fossil- or biobased, due to their key role in initiating the reactivity of both radically and cationically polymerized curable systems. Generally, radical polymerization initiators absorb electromagnetic radiation across various wavelength intervals, which transforms them into radicals with unpaired, highly reactive valence electrons.^165^ The molecule absorbs energy from a visible or UV photon, the covalent bond is broken, and a radical with a reactive, unpaired valence electron is formed.^165^ The direct radical production occurs in Type I photoinitiators, which form radicals without additional molecular assistance (typically acylphosphine oxides (BAPO and TPO))^166^ or α-hydroxyketones (Irgacure 184 and Doublecure 184).^167^ On the other hand, Type II photoinitiators require an additional co-initiator, typically organic bases (amines), to form radicals via hydrogen abstraction. The Type II photoinitiator initially forms an unstable radical with the assistance of the base, followed by the hydrogen transfer from the activated co-initiator to produce an active radical and stabilize the base co-initiator eventually.^168^ Commercial Type II photoinitiators represent benzophenone (BP), isopropyl thioxanthone (ITX), and camphorquinone (CQ).^169^ Cationic polymerization initiators generate strong acids capable of initiating epoxy ring opening, followed by aliphatic ether formation.^170^ Representative cationic initiators are aryl sulfonium salts (Cyracure, Doublecure 1176/1190) and iodonium salts.^171^ Although all of the mentioned photoinitiators are efficient, widely used, and high-performing, their chemical origin typically bears considerable fossil-based and also environmental and health hazards.^172^ Due to their reactivity and chemical structure, many applications consider restriction of their usage (such as commercial 3D printing or the nail industry) due to the new findings regarding their health-hazardous character.

Based on general and TRL 1-level research, numerous biobased chemical structures that absorb electromagnetic irradiation and form radicals for potential use in photocuring applications were reported, summarized, and investigated. Several comprehensive reviews and summaries report that many compounds exhibit suitable structural and chemical properties, including riboflavin, coumarin, flavone, curcumin, chalcone, vanillin derivatives, dihydroxyanthraquinone, naphthoquinone, β-carotene, and chlorophyll.^173–175^ However, since the Type I photoinitiator, widely used for 3D printing, is practically impossible to find in natural sources (sunlight irradiation would decompose any potential structure), all of the aforementioned biobased chromophore-bearing structures are mostly Type II photoinitiators, requiring an assisting co-initiator for functioning.^173^ Particular investigations verified the activity of listed molecules from the literature. Batchelor et al.^176^ reported that riboflavin (vitamin B2) and its mononucleotides are visible-light photoinitiators for PEG-based hydrogels. The used photoinitiator provided sufficient gelation in the investigated reactive systems but suffered considerable photodegradation during the process. Oliviera et al.^177^ demonstrated photoinitiation by curcumin and glycerol in urethane dimethacrylate (see Figure 10). Systems comprising a Type II photoinitiator and curcumin typically involve tertiary amines as efficient co-initiators; however, the authors investigated substituting these molecules (which are mostly fossil-based) with glycerol. The authors succeeded in their experimental efforts, achieving an average conversion of 53.6% for eight studied polymers. The studies investigated the activity of biobased photoinitiators but lacked a specific application. Abdallah et al.^178^ reported coumarine-based photopolymers co-initiated by iodinium salts and amines for demonstrative 3D printing. They studied the free-radical polymerization of reactive methacrylate systems, including bisphenol A-glycidyl methacrylate (BisGMA), triethylene glycol dimethacrylate (TEGDMA), 2-(hydroxyethyl)­acrylate (HEA), and trimethylolpropane triacrylate (TMPTA). The authors successfully applied the prepared initiation systems to produce 3D-printed objects, water-soluble hydrogels, and photocomposites.

**Figure 10:**
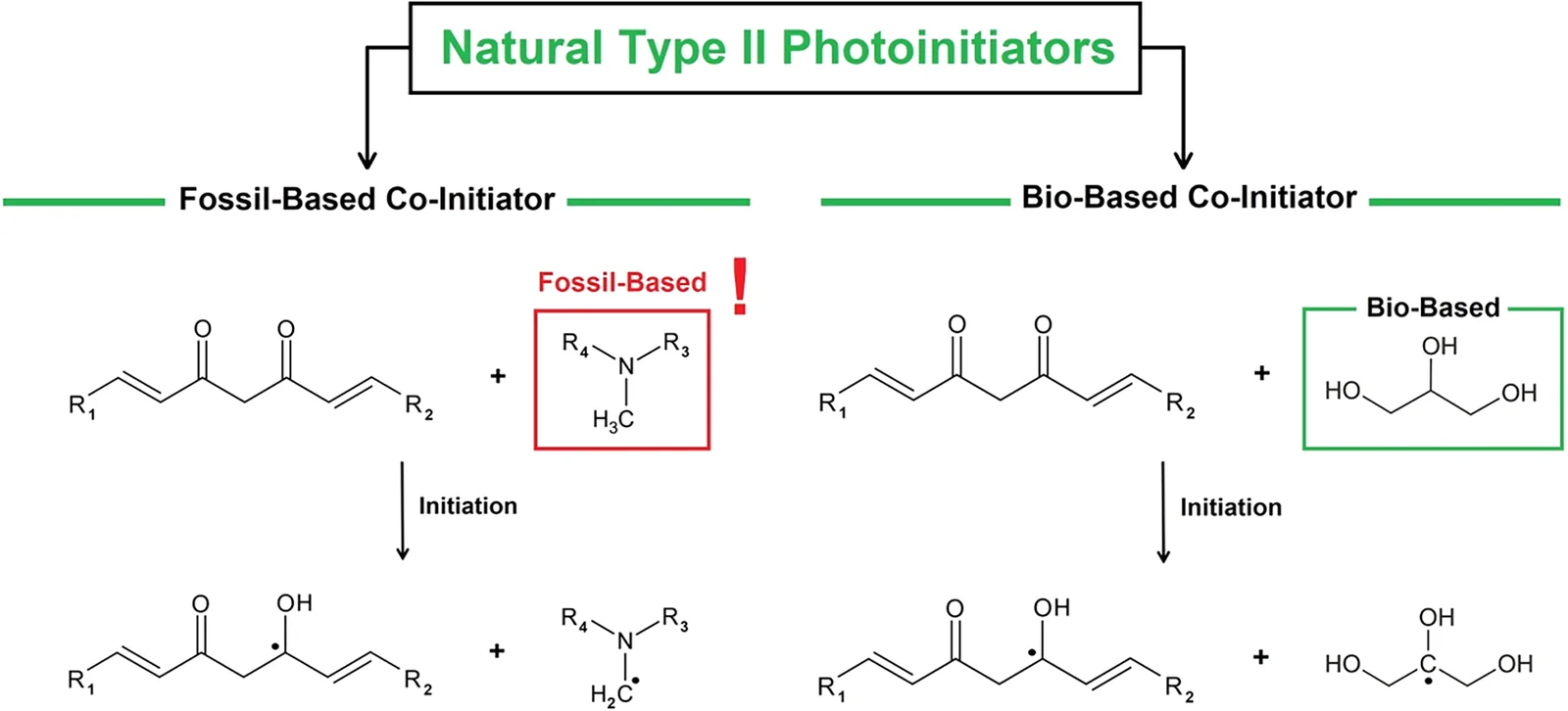
Fully biobased photoinitiator for free radical polymerization based on curcumin photosensitive compound co-initiated by tertiary amine and biobased glycerol. The substitution of traditional amine co-initiators with glycerol in photocuring applications is crucial, as it aligns with the overall biobased character of all essential components (main precursors, reactive diluents, and initiating system).^177^

The health, safety, and overall handling of biobased compounds exemplify significant potential for commercial vat photopolymerization. Since 3D printers are used by customers who do not own a hood or appropriately equipped spaces for volatile and dangerous substances, lower-hazardous systems avoid the need for additional hardware solutions, overcoming these issues in the application sector. Since the currently used photoinitiators undergo extensive testing to assess their potential adverse effects on human health, greater investigation of biobased photoinitiators is desired. However, as we reported, the majority of safer, biobased reactive initiators require multiple coreactants and are much more complicated from a straightforward, usable standpoint. Moreover, the exhibited reactivity is usually considerably lower than that of high-performing traditional systems.^104^ Apart from the increased working times and generally slower usability, homogenization issues may arise due to the differing chemical structures of the 3D printing resin and the biobased photoinitiator. We reported the fully biobased photoinitiator derived from curcumin and glycerol. The specific, highly viscous, and strictly nonpolar photocurable precursors could pose problems with the quantitative dissolution of the photoreactive initiators. Molecular engineering must consider not only functional properties but also applicability. Since biobased photoreactive compounds can be structurally modified, specific chemical tailoring may be required before the target application.

The engineering of fully biobased photoinitiators is a complex endeavor currently under extensive investigation, mainly due to health and environmental considerations. We summarized the primary points regarding the chemical structure, molecular tailoring strategy, and the eventual suitability for vat photopolymerization. The points are listed below:

- **Reactivity issues and photoinitiator type:** Since most fully biobased photoinitiators belong to the Type II photoinitiation group, their reactivity is generally lower than that of Type I photoreactive compounds. Selecting a suitable, reaction-boosting co-initiator is essential to increase the application potential of such substances. Glycerol was reported as a representative of this type of compound.
- **Solubility in photocurable resins:** Biobased photoreactive compounds exhibit various types of chemical structure. Their functional groups must be appropriately modified to ensure sufficient solubility in typically nonpolar, highly viscous resins. Esterification, acylation, or etherification represent particular modifications to less polar carbon backbones.
- **Absorption wavelength interval:** The structural modifications are essential not only from the solubility standpoint but also due to the functional absorption properties of the used photoreactive initiators. Due to the generally low-energy visible-light absorption, a specific hypsochromic shift should be achieved to shift the absorption maximum toward higher-energy photons (generally applied for Type I photoinitiators, around 400 nm). Additionally, the structural tailoring of extramolecular chromophores, which provides a hyperchromic shift in the absorption spectra of biobased photoinitiators, enhances reactivity.

## 5 Conclusion and Future Outlook

This work outlines the current trends in biobased additive manufacturing, motivated by rising societal demand for environmentally friendly, safe photocurable systems for both commercial and professional applications. Many currently used highly performing systems are fossil-based acrylates/methacrylates, diluted with volatile or petroleum-derived low-molecular-weight structures, and photoinitiated by hazardous artificial molecules. We compared the currently applied printable systems with the investigated partly- and fully biobased resins, evaluated differences in reactivity, rheology, and functional properties, and summarized the most promising options for the potential transformation of the used systems. Based on our thorough evaluation and description, we presented entirely biobased alternatives for 3D printing across all three focused branches. The brief outline of such systems is listed below (see Figure 11):

- **Photocurable resins:** Systems achieving a fully biobased composition suitable for environmentally friendlier additive manufacturing are based on photocurable, highly viscous **polyester structures** or heterogeneous reactive **terpene-itaconate systems**.
- **Reactive diluents:** Low-molecular-weight compounds, exhibiting photocurable activity and sufficiently low viscosity while maintaining optimal mechanical and functional properties, can be produced from **itaconate mono- and diesters**. Moreover, a specific **biophenolic-based** reactive diluent, synthesized from **ferulic acid**, has been reported in the literature.
- **Biobased photoinitiators:** Commercial photoinitiators are widely used in most reported cases, even in fully biobased photocurable systems, due to their straightforward functional and convenient performance. Mostly, fully biobased biosensitizers contain **riboflavin, coumarin, curcumin**, and other molecules with suitable chromophores.

**Figure 11:**
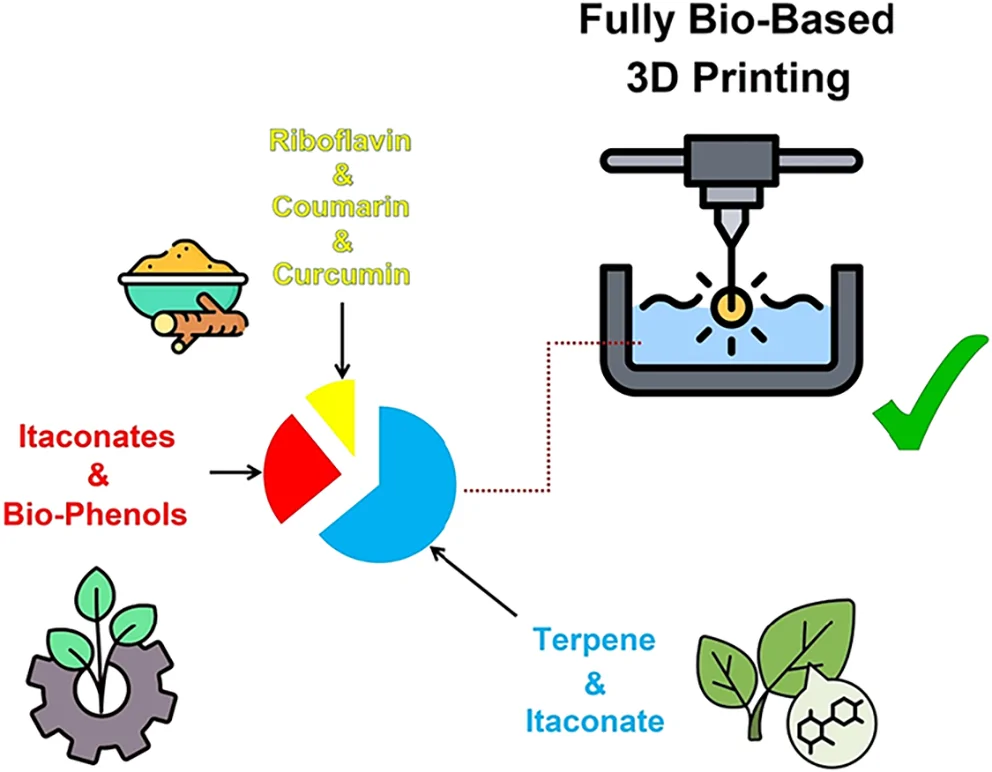
Illustration of the fully biobased opportunities toward biobased 3D-printing systems.

Considering all of the reported, summarized, and listed properties and demands, several specific outcomes leading to promising, optimized systems are concluded. The main photocurable resin should be kept at the lowest possible viscosity to avoid complicating the vat photopolymerization process because of its high viscosity (3D printers have processing limitations). So the main photocurable resins should not contain polar or hydrogen-bond-forming functional groups or excessive branching. Ideally, a short- to medium-length carbon backbone is recommended with multiple polymerizable functional groups. Reactive diluents should be highly reactive and have as low viscosity as possible to overcome the limitations of the main precursors and enable processing via 3D printing. The combination of aliphatic, structurally well-performing, and reactivity-boosting functional groups is essential. Since reactive diluents are typically low-molecular-weight compounds, the overall reactivity may be enhanced by specific polar functional groups while ensuring a sufficiently low viscosity. This compromise must be maintained primarily to provide 3D printability. The biobased photoinitiators must first fulfill the solubility requirements, which is the most essential. Without dissolution of the photoreactive compound, the functional aspect cannot be investigated. In addition to solubility, the tailored structural modifications must align with the molecular engineering of the biobased photoreactive compounds. Several absorption spectral shifts may be necessary for sufficient vat photopolymerization. Since most fully biobased photoreactive compounds belong to the Type II photoinitiator group, a suitable co-initiator must be selected to enhance the generally lower photoinitiating performance of such compounds.

Assuming biobased entering compounds as the primary driver of increased sustainability is problematic, especially given the synthetic strategies and the complex reactant and catalyst compositions in the reactions. It should be noted that even though fully biobased systems are formed, such as oligoester itaconates, the high reaction temperatures for the Fisher esterification, the hazardous nature of acyl halides, which are problematic reactants, and the use of dangerous and volatile supporting solvents negatively affect the ultimately produced precursor. The systems may comprise a biobased carbon backbone, but their overall life cycle assessment, lacking an end-of-life perspective and hazardous production processes, would not result in a more sustainable option than the fossil-based precursors. Naturally, when new concepts and materials are engineered with the intention of enhanced sustainability, alternative routes to petroleum-based systems are expected to be more energy demanding. Nevertheless, ideally, available and safe reactants, affordable and nontoxic catalysts, and minimal supporting solvents and additives should be employed in strategies toward sustainable production.

This overview provided suggestions for alternative, biobased, and environmentally friendly solutions in additive manufacturing. Naturally, many issues connected to the general synthesis of the working systems (such as complicated chemical apparatuses, the usage of supporting solvents), functional performance of the produced resins (particularly poor mechanical properties and insufficient rheological profile), and the up-scaling complications (involving high costs of the starting materials and their unavailability in industrial quantities) must be debated and addressed. Nevertheless, as this elaborate presentation and description demonstrate, several strategies and approaches have been developed; thus, various starting points for green additive manufacturing based exclusively on biobased sources have been established.

Since this Perspective is entitled with a concrete question, whether vat photopolymerization resins can consist exclusively of biobased molecules, the answer is yes, but several limitations and specific conditions must be obeyed. Standard irradiation sources in 3D printers may expose biobased resins to photons at 405 nm, which are too low in energy, leading to longer irradiation times or switching to a source with a lower wavelength and higher irradiation power. The reactivity limitations are caused by resin absorption, the lower mobility of oligomer or polymer precursors, and alternative curing strategies to radical polymerization (such as cationic polymerization of epoxy systems and thiol–ene reactions). The published working speeds are usually much higher (22–100 and more seconds per layer) compared to the commercial fossil-based systems (3–10 s per layer). The longer processing times must be considered when biobased systems are incorporated, with low-molecular-weight systems preferred that exhibit high polymerization functionality. The volatility and potential health and environmental hazards are commonly lower for biobased systems due to their typically higher viscosity and the structural character of their monomers. This aspect exemplifies the greatest added value over commercial systems, which often exhibit fewer irritants and a lower volatility. The long-term stability also poses minimal issues considering biobased resins due to their lower reactivity. Lastly, the potentially higher cost must be considered alongside the biobased alternatives, given the often energy-intensive synthesis and the cost of the entering compounds.
